# Supplementary material for: Pd(II)/Pd(IV) redox shuttle to suppress vacancy defects at grain boundaries for efficient kesterite solar cells
Source: Nat Commun. 2024 May 21;15:4344. doi: 10.1038/s41467-024-48850-9 (PMC11109278; doi:10.1038/s41467-024-48850-9)
Supplement: Supplementary file 1 — Supplementary Information [file 41467_2024_48850_MOESM1_ESM.pdf]

Supplementary information for

Pd(II)/Pd(IV) redox shuttle to suppress vacancy defects at grain boundaries for efficient kesterite solar cells

Jinlin Wang[1,2]†, Jiangjian Shi[1]†, Kang Yin[1,2]†, Fanqi Meng[3]†, Shanshan Wang[4], Licheng Lou[1,2], Jiazheng Zhou[1,2], Xiao Xu[1,2], Huijue Wu[1], Yanhong Luo[1,2,5], Dongmei Li[1,2,5]*, Shiyou Chen[4]*, and Qingbo Meng[1,5,6]*

[1]Beijing National Laboratory for Condensed Matter Physics, Institute of Physics, Chinese Academy of Sciences (CAS); Beijing, 100190, P. R. China.

[2]School of Physical Sciences, University of Chinese Academy of Sciences; Beijing, 100049, P. R. China.

[3]School of Materials Science and Engineering, Peking University; Beijing, 100871, P. R. China.

[4]School of Microelectronics, Fudan University; Shanghai, 200433, P. R. China.

[5]Songshan Lake Materials Laboratory; Dongguan, 523808, P. R. China.

[6]Center of Materials Science and Optoelectronics Engineering, University of Chinese Academy of Sciences; Beijing, 100049, P. R. China.

*Corresponding author. Email: dmli@iphy.ac.cn; chensy@fudan.edu.cn; qbmeng@iphy.ac.cn.

†These authors contributed equally to this work.

Table of contents

Supplementary Notes 1-2……………………………………………………………………..3

Supplementary Figures 1-27…………………………………………………………….4-30

Supplementary References………………………………………………………………30

Supplementary Notes

Supplementary Note1

$PdCl_2$ powder were placed in a graphite box containing Se particles, and subsequently selenized at different temperatures (350, 400, 450 and 500 °C) in a rapid heating tube furnace. The detailed selenization condition was as followed: the temperature was raised to 350, 400, 450 and 500 °C respectively within 1 min and maintained for 19 min, then reduced naturally to room temperature. XRD measurement was conducted on the $PdCl_2$ powder subjected to the selenization reaction. According to the XRD results (Fig.4c1 and Supplementary Fig. 27), $PdCl_2$ began to react with Se atom in Se atmosphere at 350 °C.

Supplementary Note 2

A mixture of 0.2 g $PdCl_2$ powder and 0.5 g Se powder (99+%, Alfa) was prepared, which was subsequently reacted at a temperature of 500 °C for a duration of 30 minutes in a tube furnace to yield $PdSe_2$. The synthesized $PdSe_2$ powder was then amalgamated with SnSe powder (99.999%, Alfa) in a mass ratio of 1:2. This composite mixture underwent further reaction in the tube furnace at a temperature of 540 °C for a period of 20 minutes. XRD analysis was conducted on the powder both pre and post the reaction process. Notable differences were observed in the XRD spectra of the mixed powder before and after the reaction process, as depicted in Fig. 4c2.

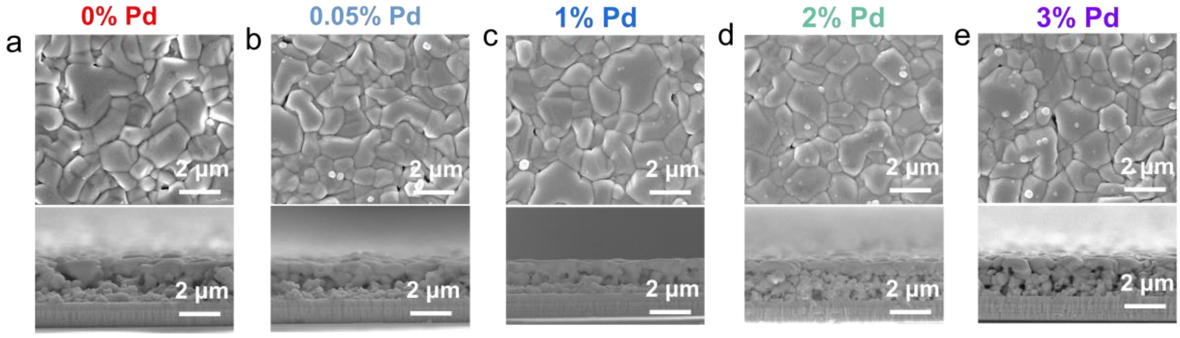

Supplementary Figure 1. The top-view and cross-sectional SEM images of ACZTSSe absorber with different Pd concentrations:(a) 0% Pd, (b) 0.05% Pd, (c)1% Pd, (d) 2% Pd, (e) 3% Pd.

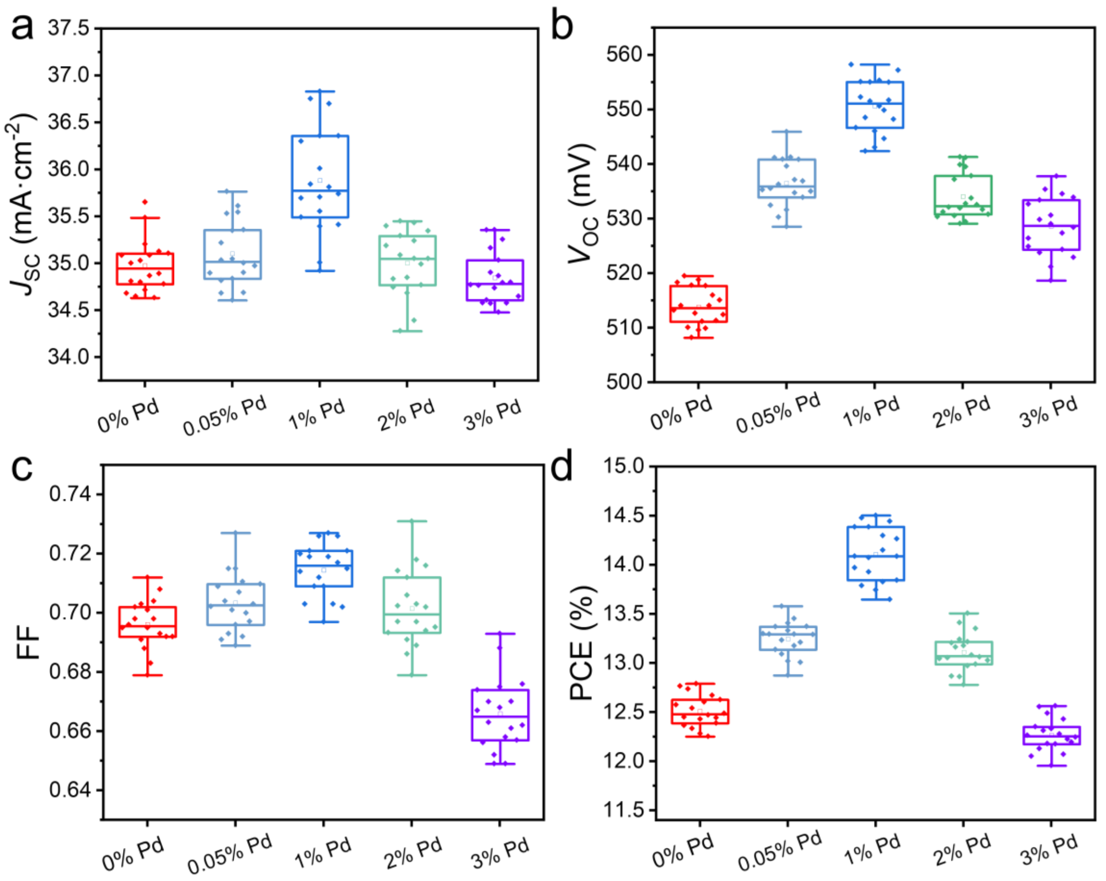

Supplementary Figure 2. Statistics performance parameters analysis of the device with different Pd concentrations: (a) J_{SC}, (b) V_{OC}, (c) FF, (d) PCE. Each box contains 18 solar cells.

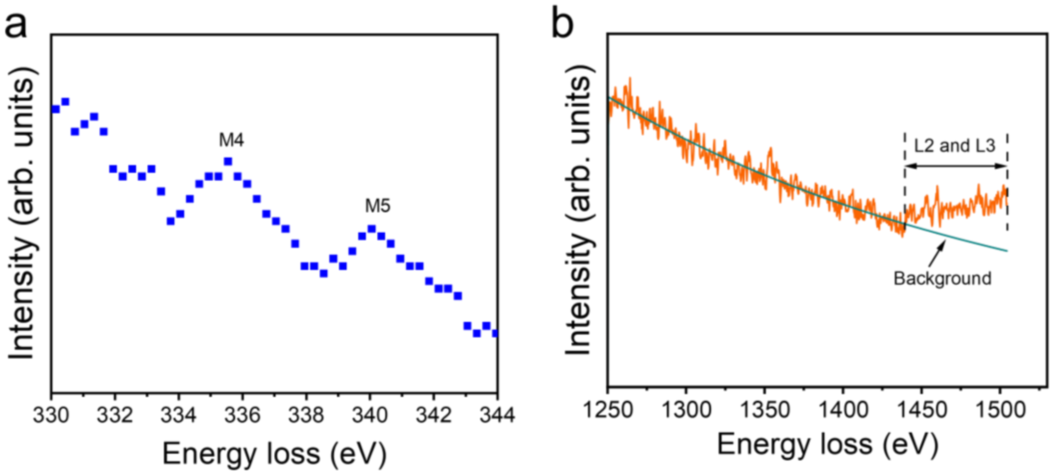

Supplementary Figure 3. Experimental low-loss EELS spectra of (a) Pd and (b) Se acquired from ACZTSSe-Pd film.

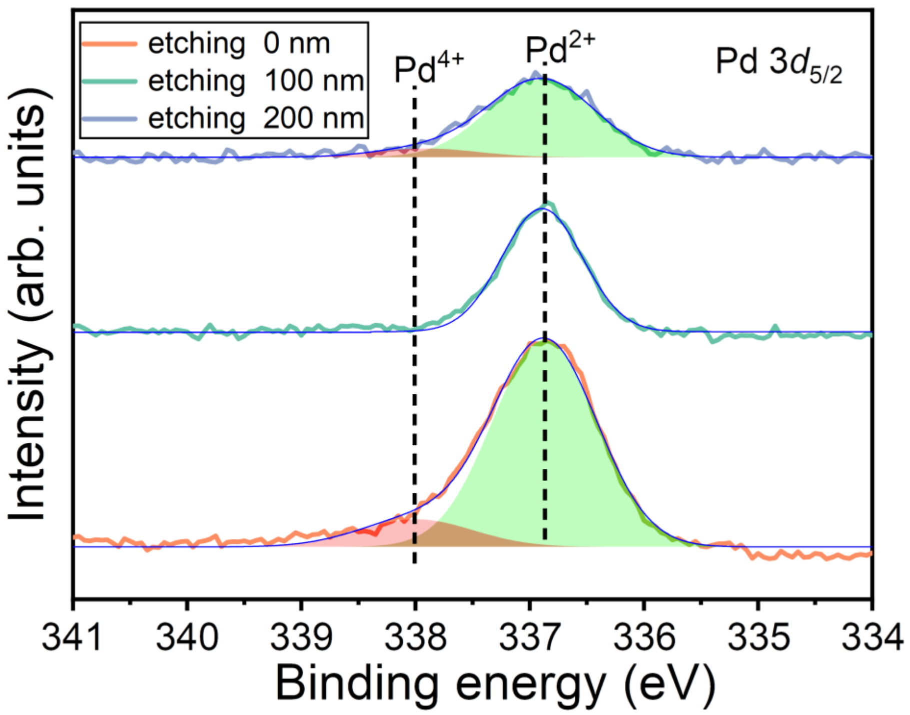

Supplementary Figure 4. XPS core level spectra of Pd $3d_{5/2}$ in ACZTSSe-Pd sample before and after argon etching. Double peak fitting was used to decouple Pd^{4+} and Pd^{2+} states.

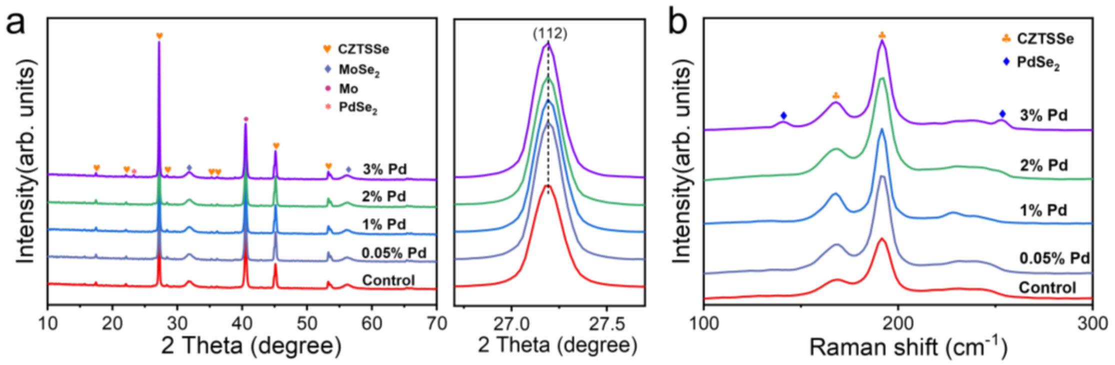

Supplementary Figure 5. (a) XRD patterns of the films with different Pd concentrations and their amplified (112) peak; (b) Raman spectra of the films with different Pd concentrations.

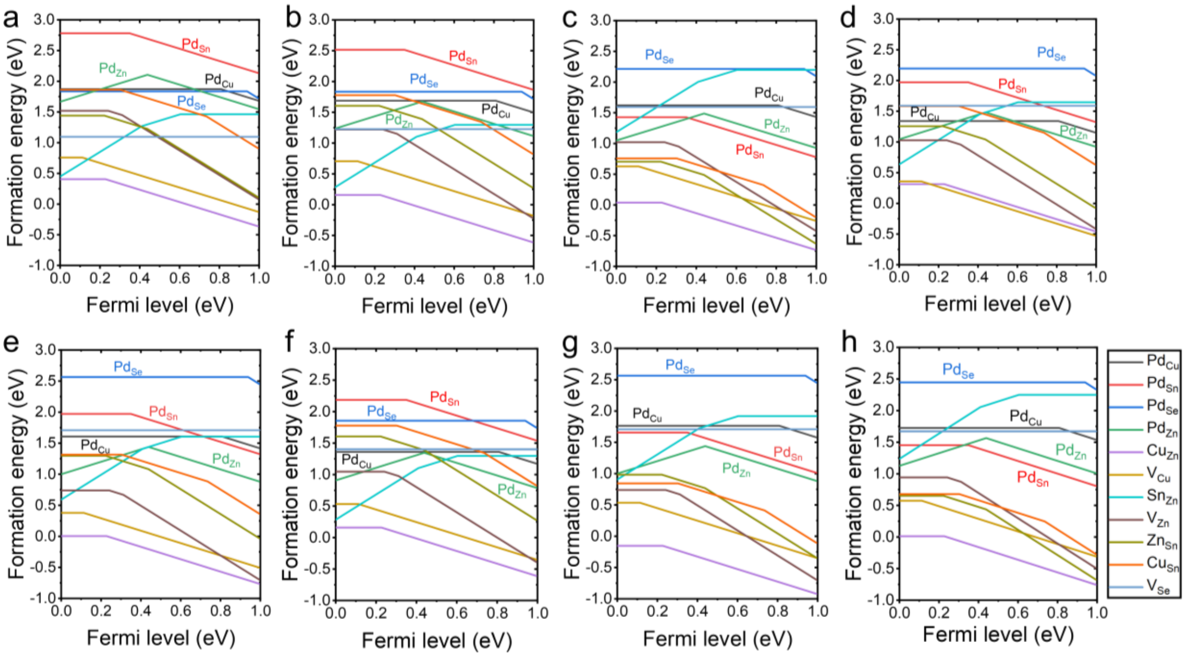

Supplementary Figure 6. The calculated formation energy of Pd dopants under the elemental chemical potential condition of (a) μ_{Cu} = -0.2007 eV, μ_{Zn} = -1.1239 eV, μ_{Sn} = -0.2559 eV, μ_{Se} = -0.6146 eV, μ_{Pd} = -1.0520 eV, (b) μ_{Cu} = -0.2507 eV, μ_{Zn} = -1.4217 eV, μ_{Sn} = -0.3885 eV, μ_{Se} = -0.4820 eV, μ_{Pd} = -0.9193 eV, (c) μ_{Cu} = -0.3307 eV, μ_{Zn} = -1.6218 eV, μ_{Sn} = -1.4896 eV, μ_{Se} = -0.1167 eV, μ_{Pd} = -0.9325 eV, (d) μ_{Cu} = -0.6000 eV, μ_{Zn} = -1.6156 eV, μ_{Sn} = -0.9324 eV, μ_{Se} = -0.1229 eV, μ_{Pd} = -0.9201 eV, (e) μ_{Cu} = -0.5788 eV, μ_{Zn} = -1.9037 eV, μ_{Sn} = -1.1782 eV, μ_{Se} = 0.0000 eV, μ_{Pd} = -1.1657 eV, (f) μ_{Cu} = -0.4250 eV, μ_{Zn} = -1.5960 eV, μ_{Sn} = -0.5628 eV, μ_{Se} = -0.3077 eV, μ_{Pd} = -0.7654 eV, (g) μ_{Cu} = -0.4215 eV, μ_{Zn} = -1.9037 eV, μ_{Sn} = -1.4929 eV, μ_{Se} = 0.0000 eV, μ_{Pd} = -1.1657 eV, (h) μ_{Cu} = -0.3825 eV, μ_{Zn} = -1.6995 eV, μ_{Sn} = -1.6191 eV, μ_{Se} = -0.039 eV, μ_{Pd} = -1.0877 eV.

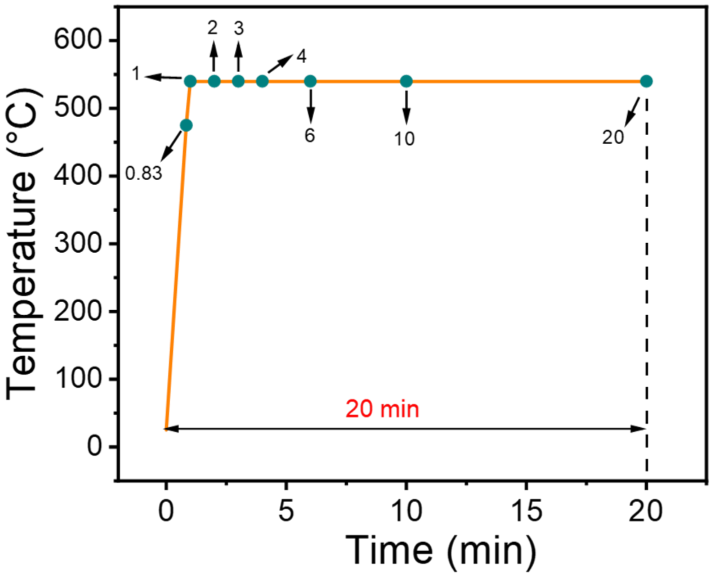

Supplementary Figure 7. The schematic temperature profile of the selenization process. The total time for optimal selenization is 20 min from room temperature. The selenization was interrupted at 0.83, 1, 2, 3, 4, 6, 10, and 20 min. For simplicity of description, samples at different stages are labelled as t, where t indicates the total selenization time from room temperature.

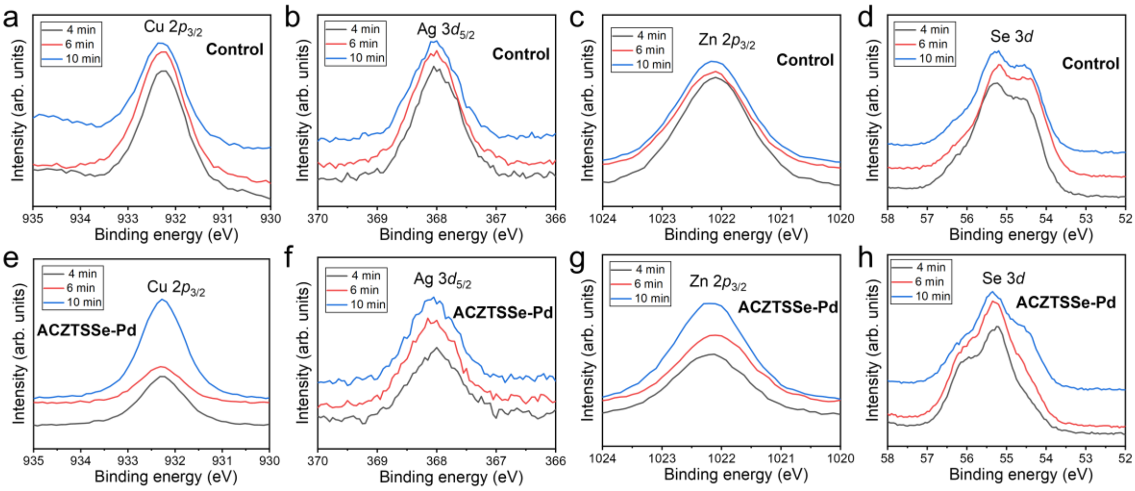

Supplementary Figure 8. XPS spectra of (a) Cu 2$p_{3/2}$, (b) Ag 3$d_{5/2}$, (c) Zn 2$p_{3/2}$ and (d) Se 3d of control films at 4, 6 and 10 min. XPS spectra of (e) Cu 2$p_{3/2}$, (f) Ag 3$d_{5/2}$, (g) Zn 2$p_{3/2}$ and (h) Se 3d of ACZTSSe-Pd films at 4, 6 and 10 min.

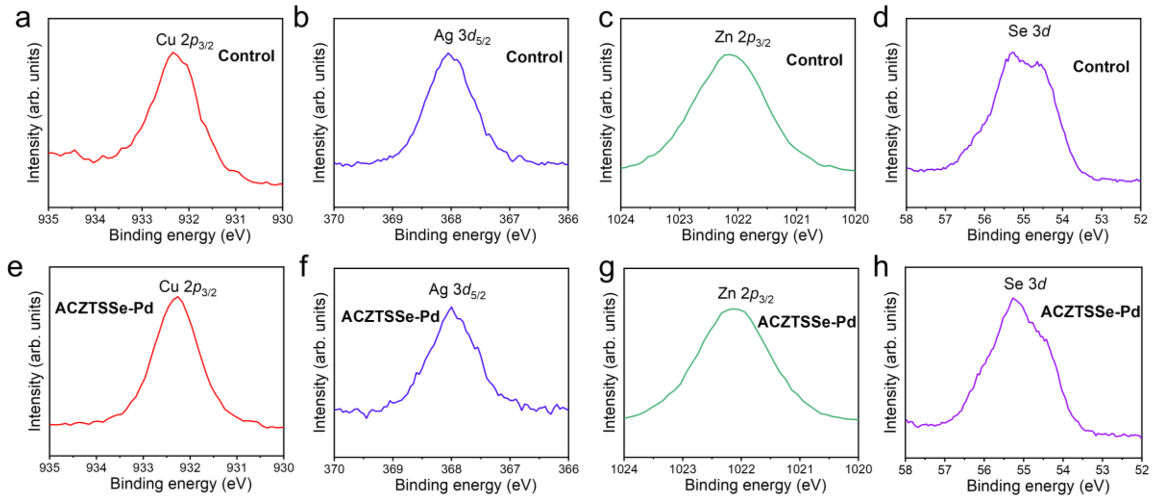

Supplementary Figure 9. XPS spectra of (a, e) Cu $2p_{3/2}$, (b, f) Ag $3d_{5/2}$, (c, g) Zn $2p_{3/2}$ and (d, h) Se 3*d* of final-state control and ACZTSSe-Pd films (a-d: Control, e-h: ACZTSSe-Pd).

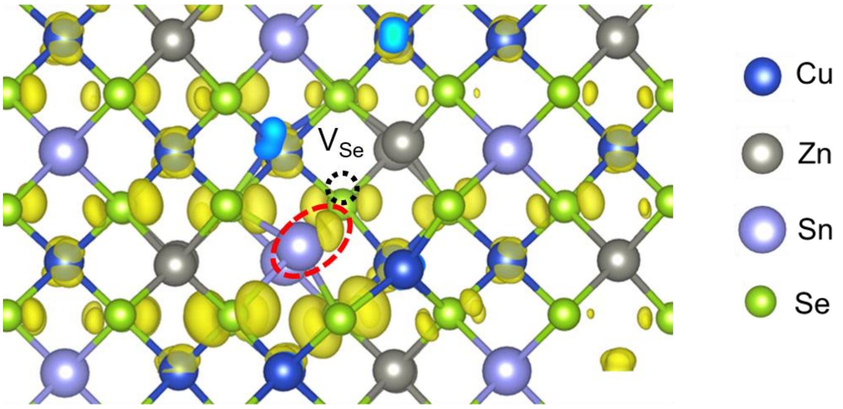

Supplementary Figure 10. Norm-squared wave function of the highest occupied eigenstate produced by V_{Se} in neutral state.

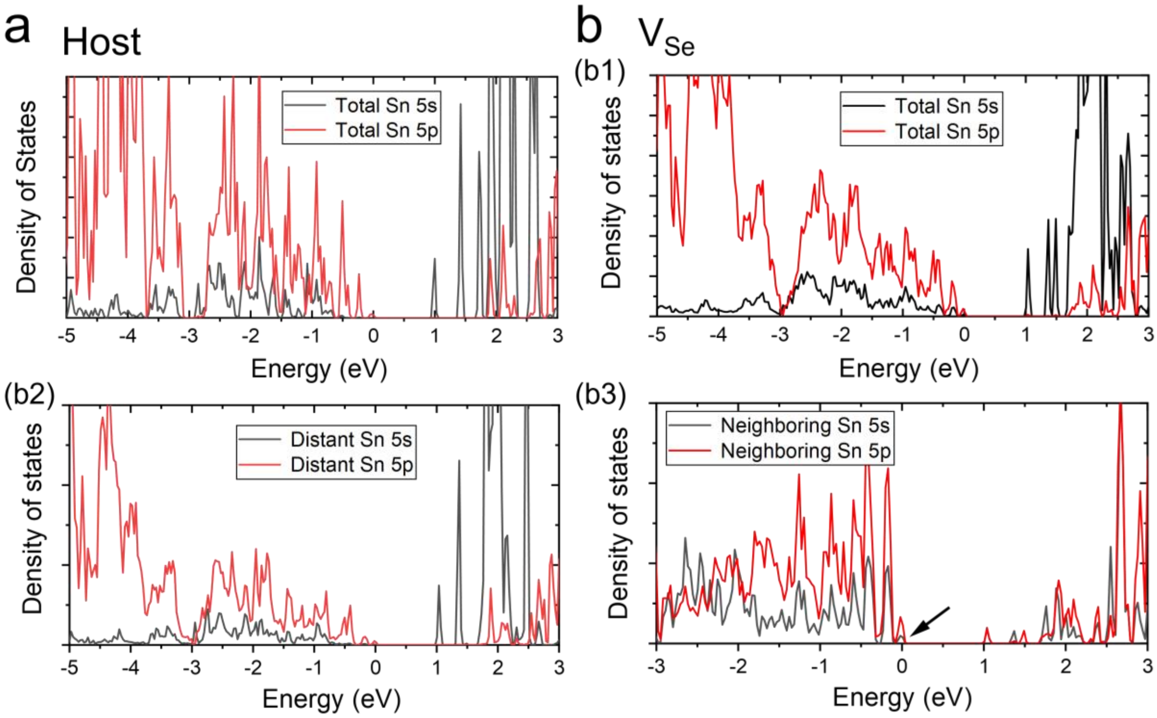

Supplementary Figure 11. Density of states diagram of the (a) host and (b) V_{Se}-containng supercells.

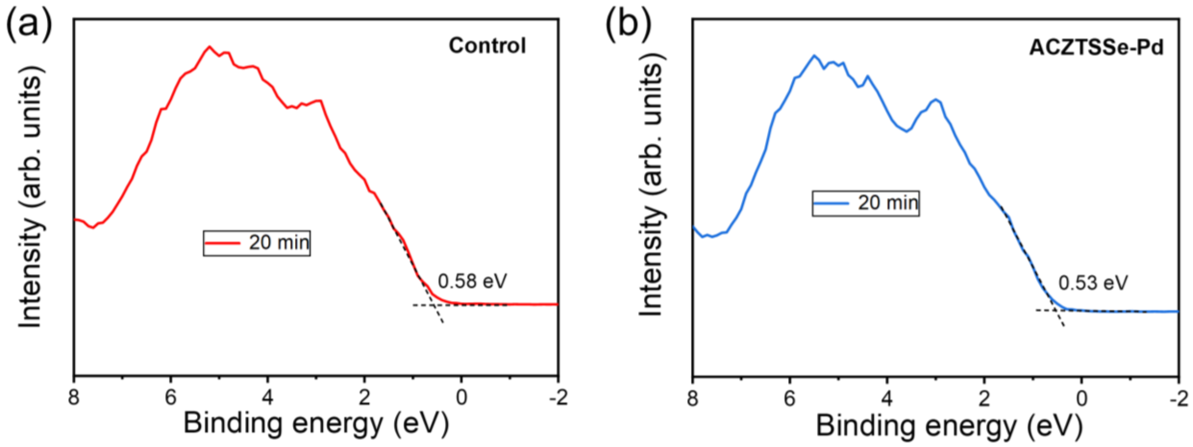

Supplementary Figure 12. The measured XPS valence-band spectra of (a) control and (b) ACZTSSe-Pd films selenized for 20 min. The dashed black lines mark the baseline and the tangents of the curve. The intersections of the tangents with the baseline give the valence band maximum position vs Fermi energy.

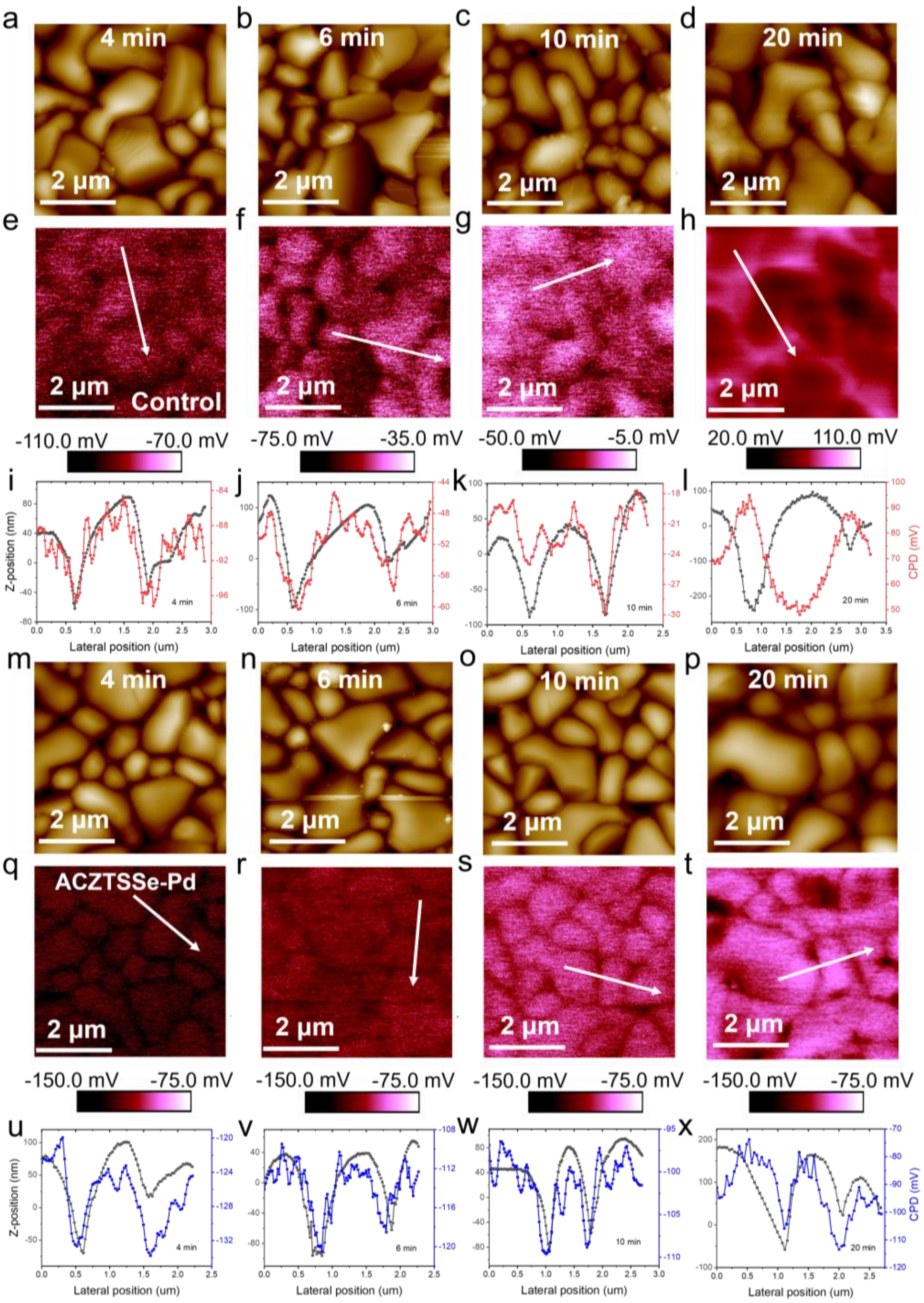

Supplementary Figure 13. AFM and KPFM images and topography and CPD line scans of control films selenized at (a,e,i) 4 min, (b,f,j) 6 min, (c,g,k) 10 min and (d,h,l) 20 min. AFM and KPFM images and topography and CPD line scans of ACZTSSe-Pd films selenized at (m,q,u) 4 min, (n,r,v) 6 min, (o,s,w) 10 min and (p,t,x) 20 min. For the control sample, in the first 10 mins selenization, lower CPD was obtained in the GB regions, similar to that of the ACZTSSe-Pd sample. However, due to the Se loss in the later-stage high temperature process, a significant amount of V_{Se} would appear in the GB regions of the control sample, introducing donor charge, thus causing the contrast inverts of the CPD in the GB regions. Comparatively, the ACZTSSe-Pd sample did not show this phenomenon because the element loss has been effectively suppressed.

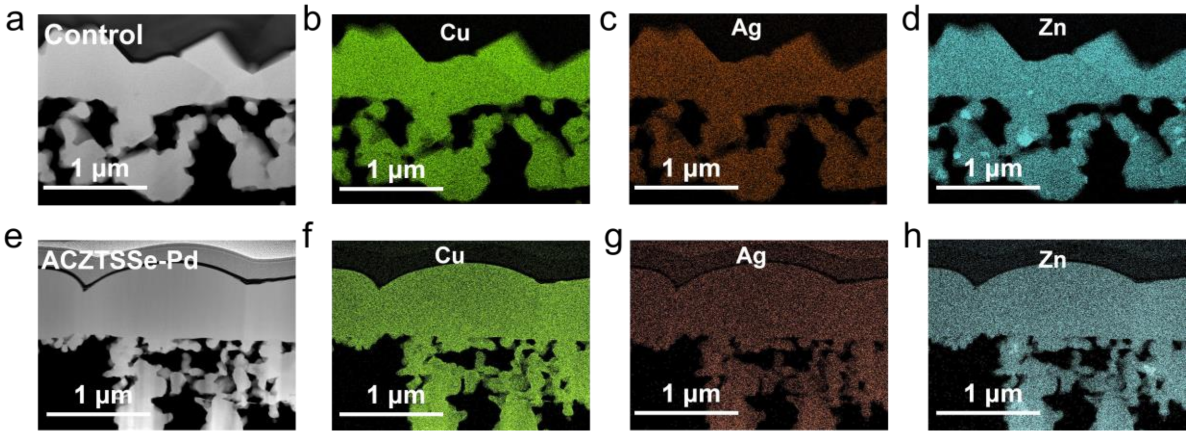

Supplementary Figure 14. (a) STEM micrograph of control film; EDS mapping of (b) Cu, (c) Ag and (d) Zn from the control film. (e) STEM micrograph of ACZTSSe-Pd film; EDS mapping of (f) Cu, (g) Ag and (h) Zn from the ACZTSSe-Pd film.

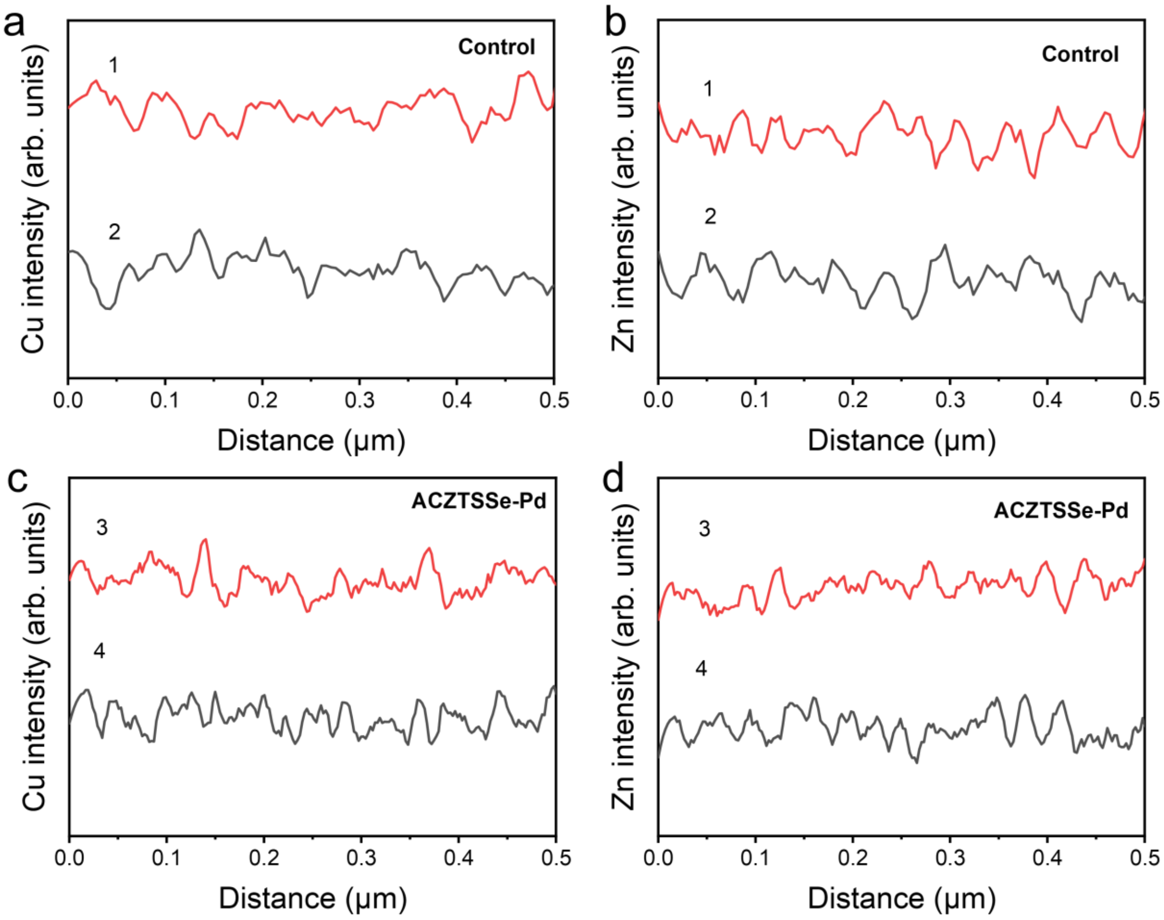

Supplementary Figure 15. The line scans of control film obtained from the EDS mapping of (a) Cu and (b) Zn; The line scans of ACZTSSe-Pd film obtained from the EDS mapping of (c) Cu and (d) Zn.

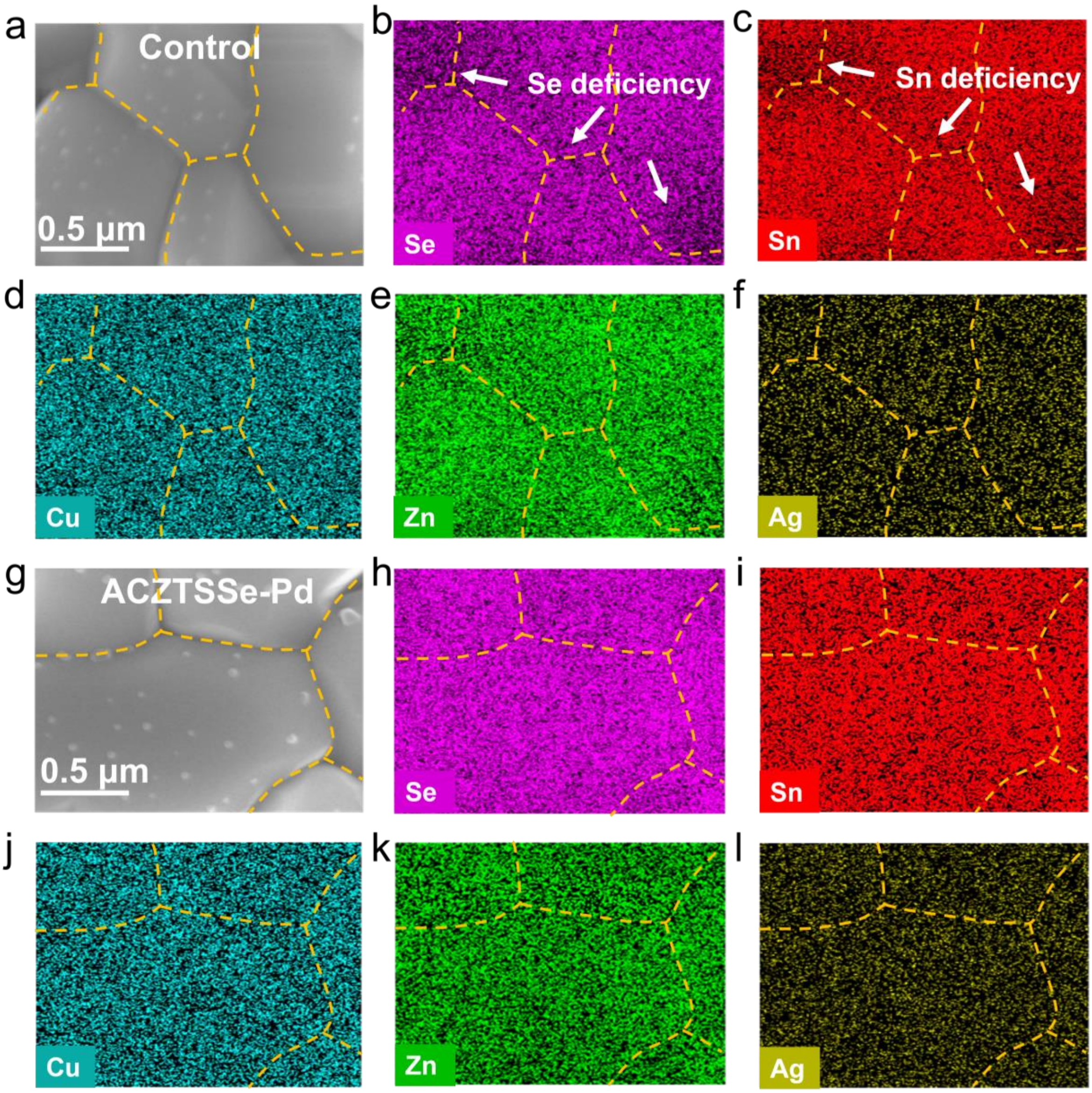

Supplementary Figure 16. (a) Top-view SEM image and (b-f) EDX mapping of the control sample (b: Se, c: Sn, d: Cu, e: Zn, f: Ag). (g) Top-view SEM image and (h-l) EDX mapping of the ACZTSSe-Pd film (h: Se, i: Sn, j: Cu, k: Zn, l: Ag). GBs are marked with yellow dotted lines. It can be qualitatively seen in (b-c) in some GB regions Se and Sn exhibited obvious deficiency, as depicted by the white arrows, while for the ACZTSSe-Pd sample in (h-i), no obvious Sn or Se deficiency was observed in the GB region.

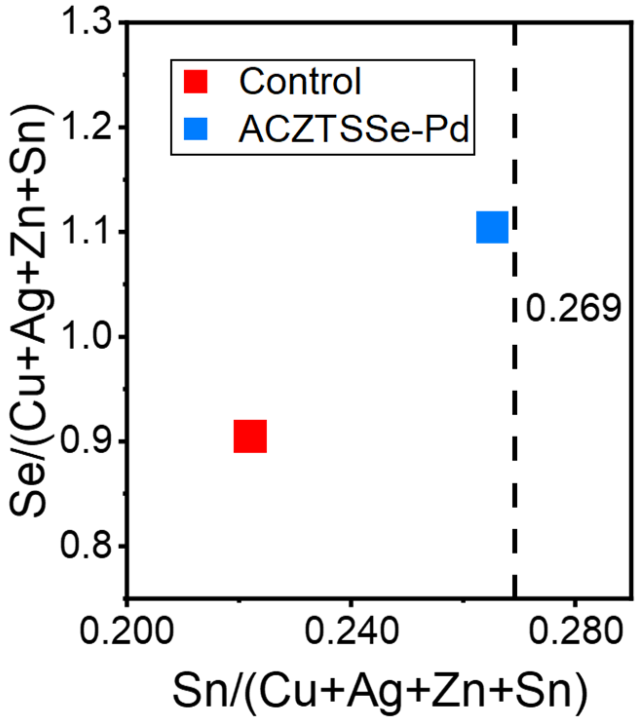

Supplementary Figure 17. Compositional ratios estimated from the SEM-EDX mappings. The vertical dotted line represents the ratio of Sn/(Cu+Ag+Zn+Sn) in the precursor solution.

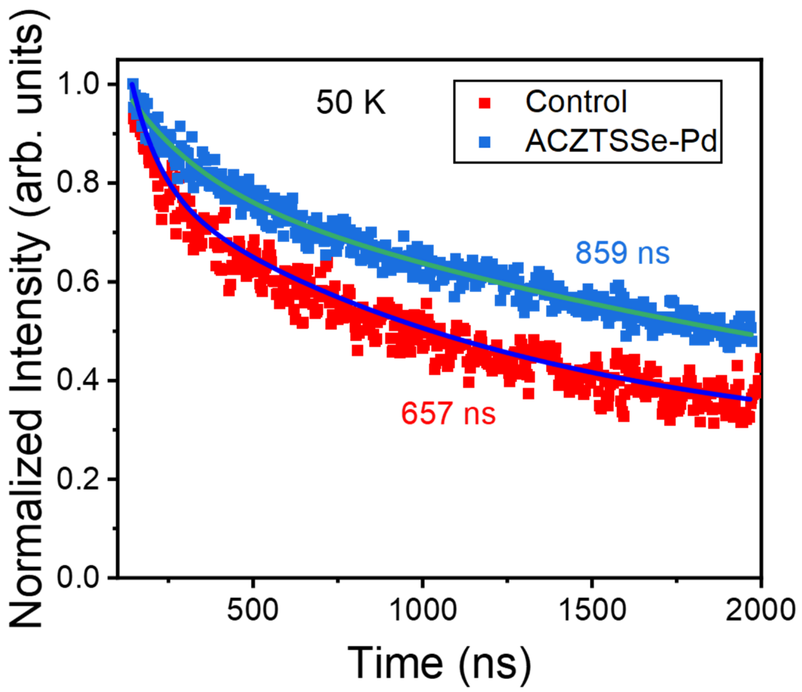

Supplementary Figure 18. Normalized time-resolved photoluminescence decay for control device and ACZTSSe-Pd device.

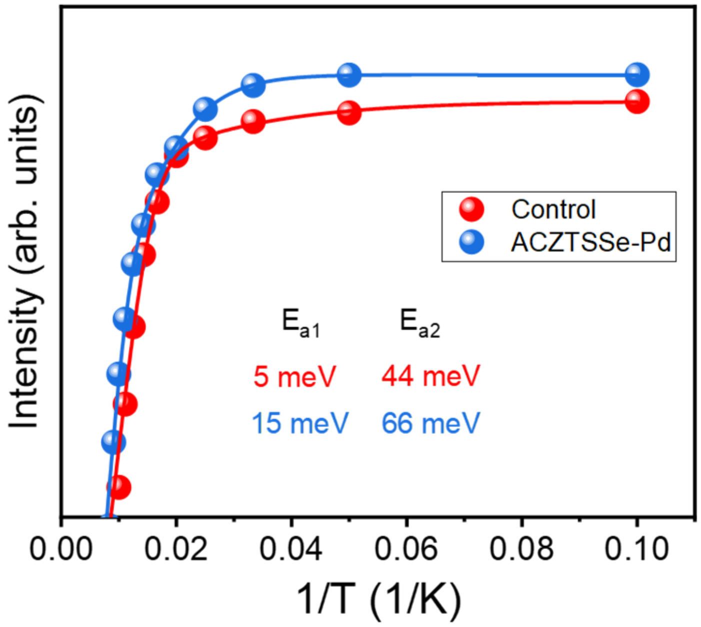

Supplementary Figure 19. PL intensity and the activation energy of PL quenching (E_{a1} and E_{a2}) estimated from steady-state PL spectra.

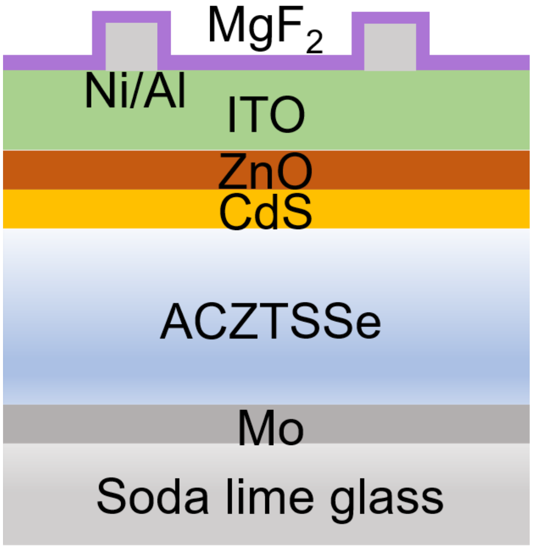

Supplementary Figure 20. Schematic structure of the ACZTSSe device.

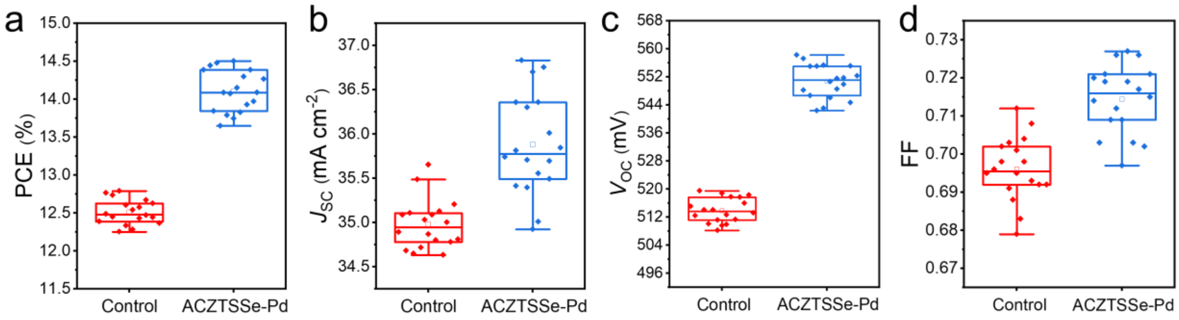

Supplementary Figure 21. Statistics analysis of the device performance parameters. (a) PCE; (b) J_{SC}; (c) V_{OC}; (d) FF. Each box contains 18 solar cells.

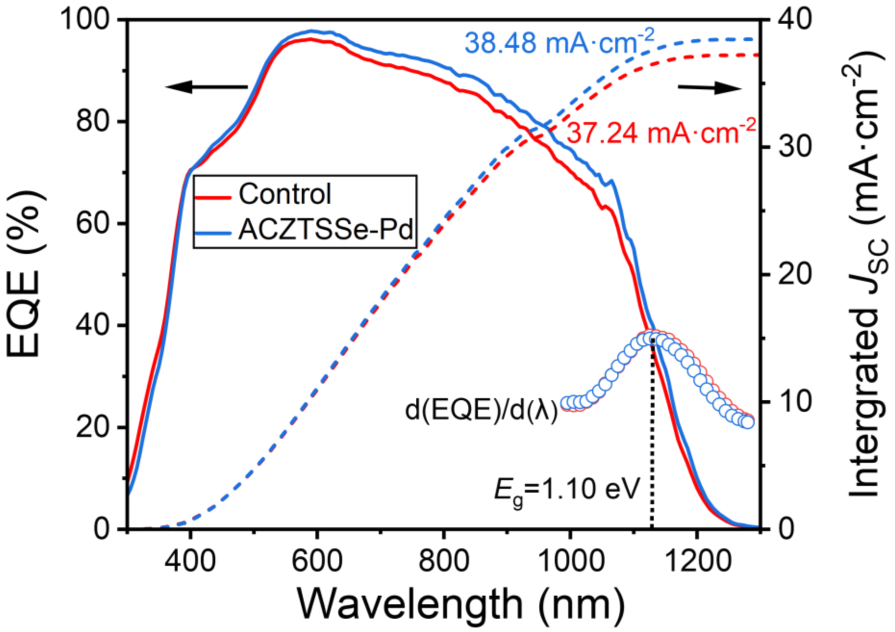

Supplementary Figure 22. EQE spectra and their current density integration of the cells. The integrated J_{SC} of the ACZTSSe-Pd cell is about 38.5 mA cm^{-2}, which agrees well with the total-area J_{SC} (36.7 mA cm^{-2}) of the cell shown in Figure 3.

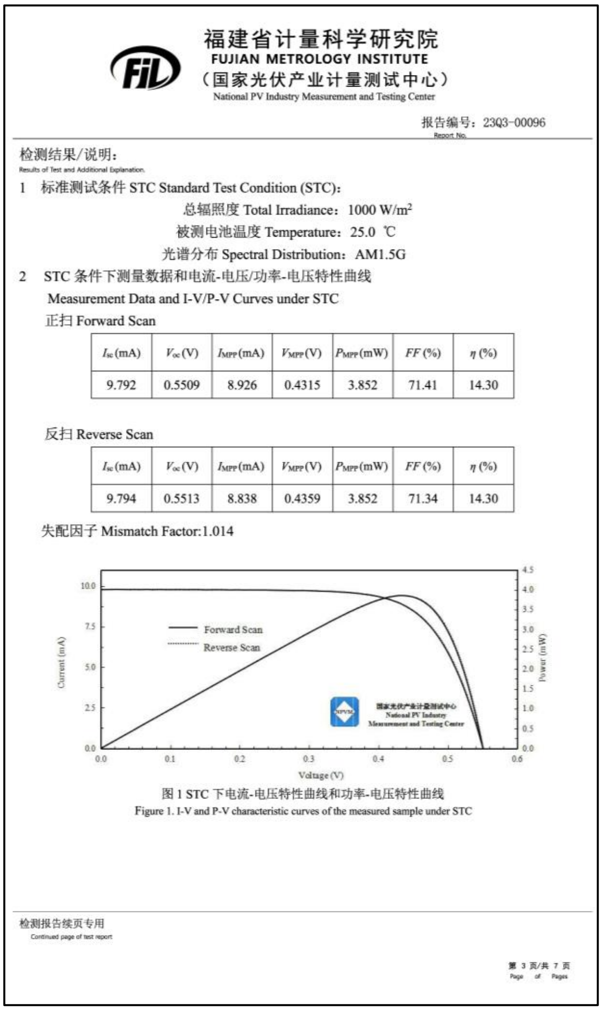

福建省计量科学研究院
FUJIAN METROLOGY INSTITUTE
（国家光伏产业计量测试中心）
National PV Industry Measurement and Testing Center

报告编号：23Q3-00096
Report No.

检测结果/说明：
Results of Test and Additional Explanation.

1 标准测试条件 STC Standard Test Condition (STC)：

总辐照度 Total Irradiance：1000 W/m^2

被测电池温度 Temperature：25.0 ℃

光谱分布 Spectral Distribution：AM1.5G

2 STC 条件下测量数据和电流-电压/功率-电压特性曲线
Measurement Data and I-V/P-V Curves under STC

正扫 Forward Scan

I_{sc} (mA)	V_{oc} (V)	I_{MPP} (mA)	V_{MPP} (V)	P_{MPP} (mW)	FF (%)	η (%)
9.792	0.5509	8.926	0.4315	3.852	71.41	14.30

反扫 Reverse Scan

I_{sc} (mA)	V_{oc} (V)	I_{MPP} (mA)	V_{MPP} (V)	P_{MPP} (mW)	FF (%)	η (%)
9.794	0.5513	8.838	0.4359	3.852	71.34	14.30

失配因子 Mismatch Factor:1.014

图 1 STC 下电流-电压特性曲线和功率-电压特性曲线
Figure 1. I-V and P-V characteristic curves of the measured sample under STC

检测报告续页专用
Continued page of test report

第 3 页/共 7 页
Page of Pages

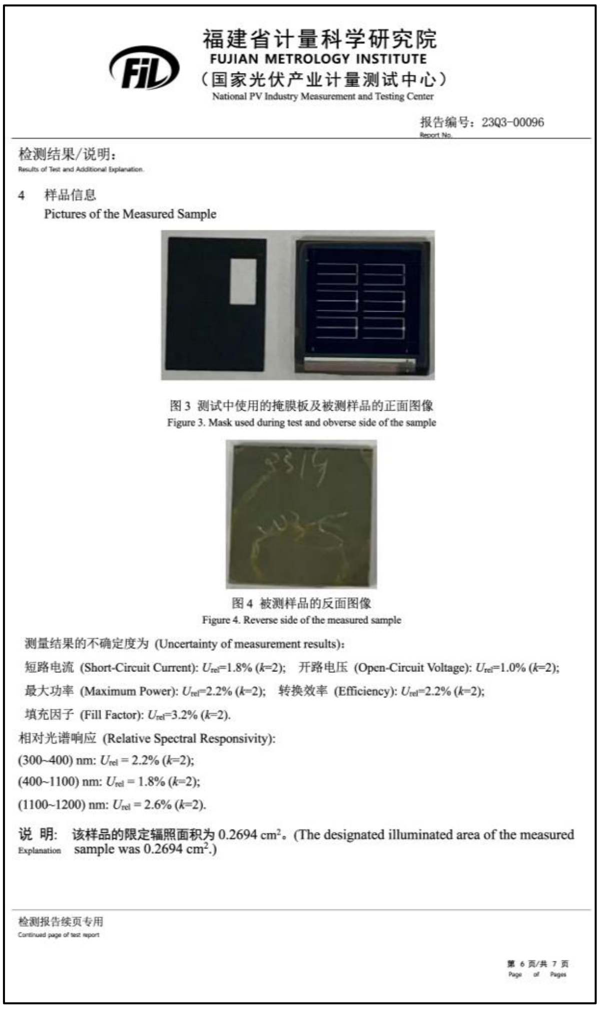

福建省计量科学研究院
FUJIAN METROLOGY INSTITUTE
（国家光伏产业计量测试中心）
National PV Industry Measurement and Testing Center

报告编号：23Q3-00096
Report No.

检测结果/说明：
Results of Test and Additional Explanation.

4 样品信息
Pictures of the Measured Sample

图 3 测试中使用的掩膜板及被测样品的正面图像
Figure 3. Mask used during test and obverse side of the sample

图 4 被测样品的反面图像
Figure 4. Reverse side of the measured sample

测量结果的不确定度为 (Uncertainty of measurement results)：

短路电流 (Short-Circuit Current): U_{rel}=1.8% (k=2); 开路电压 (Open-Circuit Voltage): U_{rel}=1.0% (k=2);

最大功率 (Maximum Power): U_{rel}=2.2% (k=2); 转换效率 (Efficiency): U_{rel}=2.2% (k=2);

填充因子 (Fill Factor): U_{rel}=3.2% (k=2).

相对光谱响应 (Relative Spectral Responsivity):

(300~400) nm: U_{rel} = 2.2% (k=2);

(400~1100) nm: U_{rel} = 1.8% (k=2);

(1100~1200) nm: U_{rel} = 2.6% (k=2).

说 明: 该样品的限定辐照面积为 0.2694 cm^2。(The designated illuminated area of the measured sample was 0.2694 cm^2.)
Explanation

检测报告续页专用
Continued page of test report

第 6 页/共 7 页
Page of Pages

Supplementary Figure 23. Certification test report from Chinese National Photovoltaic Industry Measurement and Testing Center (NPVM). The mask area (designated illuminated area): 26.94 mm^2. The total-area efficiency was 14.3%. The test report was used with the permission from NPVM.

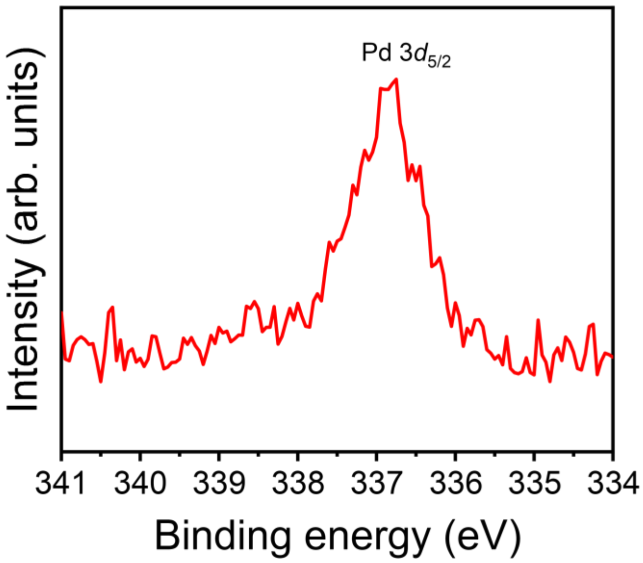

Supplementary Figure 24. XPS spectra of Pd 3$d_{5/2}$ of ACZTS-Pd precursor film.

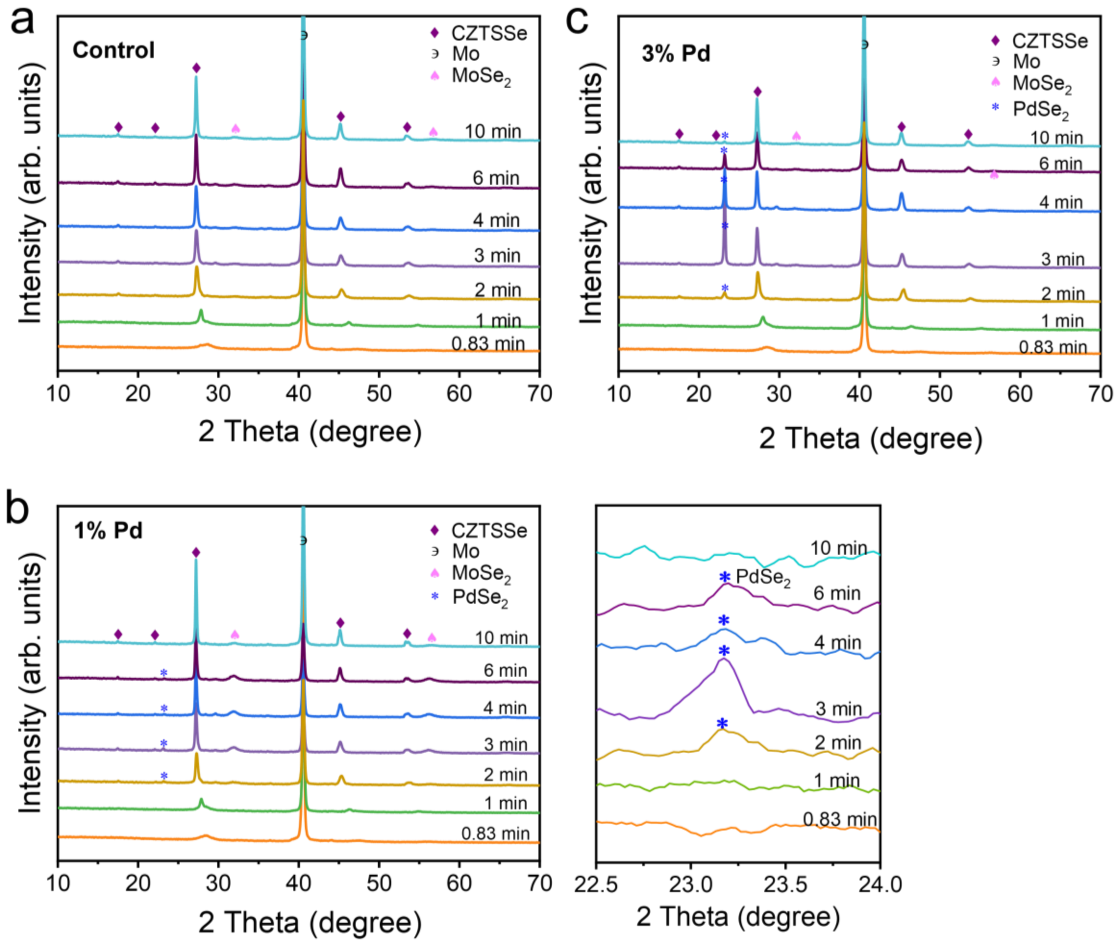

Supplementary Figure 25. XRD patterns of (a) control films and (c) ACZTSSe-3%Pd films at different selenization stages; (b) XRD patterns of ACZTSSe-1%Pd films at different selenization stages and expanded peak of $PdSe_2$ at 23.1° (PDF#11-0453).

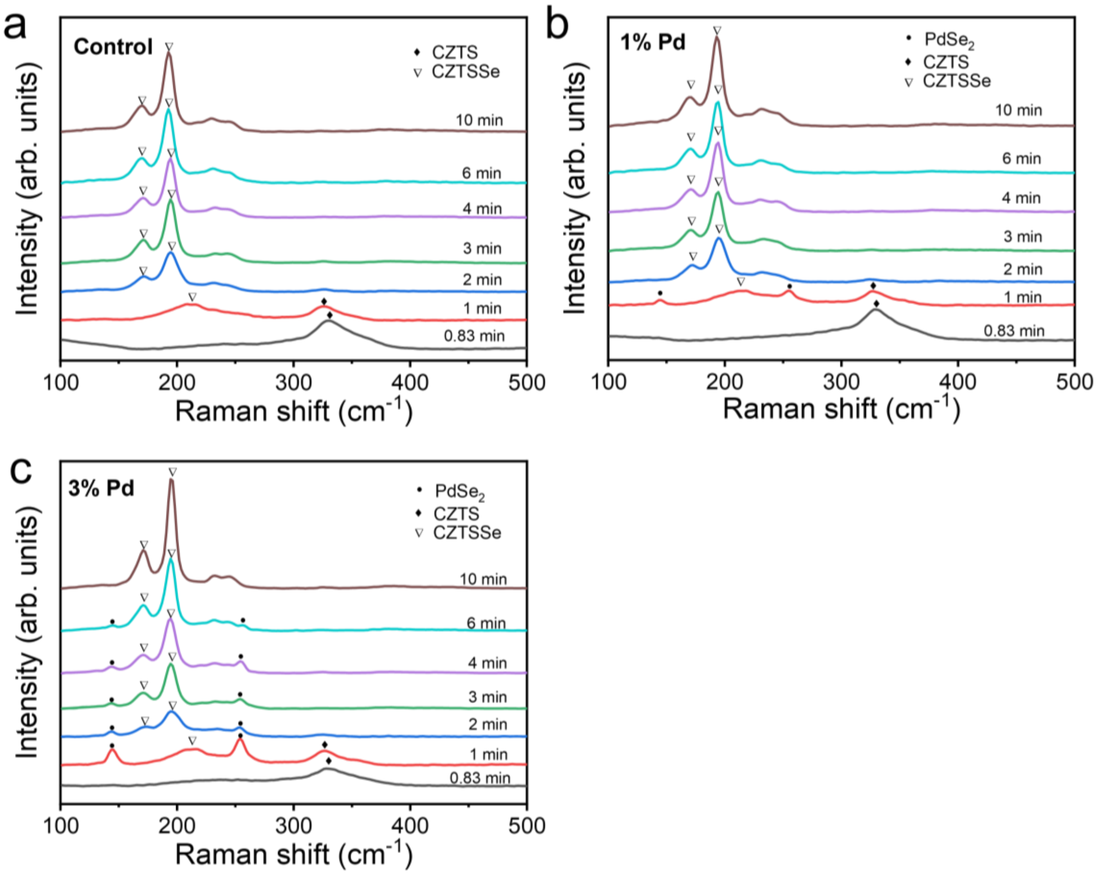

Supplementary Figure 26. Raman spectra of (a) control films, (b) ACZTSSe-1% Pd films and (c) ACZTSSe-3% Pd films at different selenization stages. Two Raman peaks with 143 and 256 cm^{-1} are attributed to $PdSe_2$.[1]

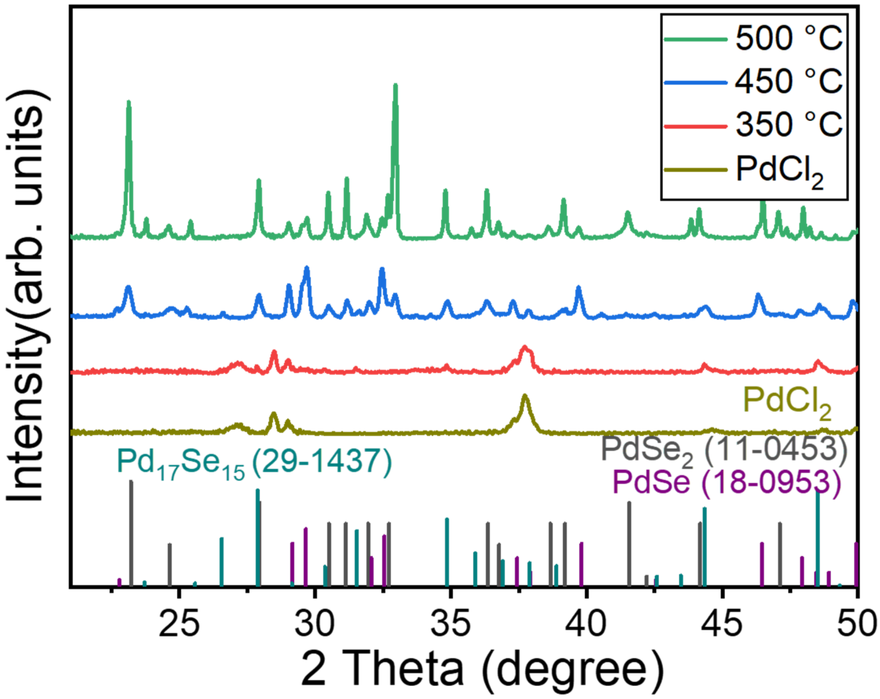

Supplementary Figure 27. XRD spectra of $PdCl_2$ before and after reaction with Se in Se atmosphere at 350 ℃,450 ℃ and 500 ℃.

Supplementary References

1 Chow, W. L. *et al.* High Mobility 2D Palladium Diselenide Field-Effect Transistors with Tunable Ambipolar Characteristics. *Adv. Mater.* **29**, 1602969 (2017).
