# Supplementary material for: Pd(II)/Pd(IV) redox shuttle to suppress vacancy defects at grain boundaries for efficient kesterite solar cells
Source: Nat Commun. 2024 May 21;15:4344. doi: 10.1038/s41467-024-48850-9 (PMC11109278; doi:10.1038/s41467-024-48850-9)
Supplement: Supplementary file 3 — Reporting Summary [file 41467_2024_48850_MOESM3_ESM.pdf]

nature portfolio

Corresponding author(s): Qingbo Meng, Shiyou Chen, Dongmei Li

Solar Cells Reporting Summary

Nature Portfolio wishes to improve the reproducibility of the work that we publish. This form is intended for publication with all accepted papers reporting the characterization of photovoltaic devices and provides structure for consistency and transparency in reporting. Some list items might not apply to an individual manuscript, but all fields must be completed for clarity.

For further information on Nature Research policies, including our data availability policy, see Authors & Referees.

Experimental design

Please check the following details are reported in the manuscript, and provide a brief description or explanation where applicable.

1. Dimensions

Area of the tested solar cells

- [x] Yes — The area is 0.2694 cm2 and it is given in experimental section and in the certification report.
- [ ] No — *Explain why this information is not reported/not relevant.*

Method used to determine the device area

- [x] Yes — The device area is determined by mask and it is given in experimental section and in the certification report.
- [ ] No — *Explain why this information is not reported/not relevant.*

2. Current-voltage characterization

Current density-voltage (J-V) plots in both forward and backward direction

- [x] Yes — The forward and backward scan J-V curves are given in certification report and it shows no hysteresis.
- [ ] No

Voltage scan conditions

- [x] Yes — The voltage was forward scanned from -50 mV to 600 mV with a scanning rate of 90 mV·s-1 and it is given in experimental section.
- [ ] No — *Explain why this information is not reported/not relevant.*

Test environment

- [x] Yes — The J-V tests were conducted in air at 25 °C. The test environment is given in experimental section.
- [ ] No — *Explain why this information is not reported/not relevant.*

Protocol for preconditioning of the device before its characterization

- [x] Yes — No preconditioning of the device was performed before the measurement. This is given in experimental section.
- [ ] No — *Explain why this information is not reported/not relevant.*

Stability of the J-V characteristic

- [ ] Yes — *Provide a description of the method used. The stability of the J-V characteristic can be verified with time evolution of the maximum power point or with the photocurrent at maximum power point; see ref. 5 for details.*
- [x] No — Kesterite solar cells do not have hysteresis . And in the certification process, maximum power output had been traced for several minutes to confirm the cell efficiency.

3. Hysteresis or any other unusual behaviour

Description of the unusual behaviour observed during the characterization

- [ ] Yes — *Provide a description of hysteresis or any other unusual behaviour observed during the characterization.*
- [x] No — Kesterite solar cells do not have hysteresis or other unusual behaviour.

Related experimental data

- [ ] Yes — *Provide a description of the related experimental data.*
- [x] No — Kesterite solar cells do not have hysteresis or other unusual behaviour.

4. Efficiency

External quantum efficiency (EQE) or incident photons to current efficiency (IPCE)

- [x] Yes — EQE is given in supplementary materials.
- [ ] No — *Explain why this information is not reported/not relevant.*

A comparison between the integrated response under the standard reference spectrum and the response measure under the simulator	☒ Yes ☐ No	The integrated JSC is about 38.5 mA/cm2, which agrees well with the total-area JSC (36.7 mA/cm2) of the cell. The comparison is given in supplementary materials.<br>*Explain why this information is not reported/not relevant.*
For tandem solar cells, the bias illumination and bias voltage used for each subcell	☐ Yes ☒ No	*Provide a description of the measurement conditions.*<br>No tandem solar cell was studied.

5. Calibration

Light source and reference cell or sensor used for the characterization	☒ Yes ☐ No	The J-V curves of the solar cells were measured by using Keithley 2400 Source Meter under simulated AM 1.5 sunlight at 100 mW cm−2 calibrated with Si reference cell. This is given in experimental section.<br>*Explain why this information is not reported/not relevant.*
Confirmation that the reference cell was calibrated and certified	☒ Yes ☐ No	The J-V curves of the solar cells were measured by using Keithley 2400 Source Meter under simulated AM 1.5 sunlight at 100 mW cm−2 calibrated with Si reference cell. This is given in experimental section.<br>*Explain why this information is not reported/not relevant.*
Calculation of spectral mismatch between the reference cell and the devices under test	☒ Yes ☐ No	The mismatch factor is 1.014 and this is given in certification report<br>*Explain why this information is not reported/not relevant.*

6. Mask/aperture

Size of the mask/aperture used during testing	☒ Yes ☐ No	The mask area is 0.2694 cm2 and this is given in certification report<br>*Explain why this information is not reported/not relevant.*
Variation of the measured short-circuit current density with the mask/aperture area	☐ Yes ☒ No	*Report the difference in the short-circuit current density values measured with the mask and aperture area.*<br>No such phenomenon in kesterite solar cells

7. Performance certification

Identity of the independent certification laboratory that confirmed the photovoltaic performance	☒ Yes ☐ No	The photovoltaic performance is certified in NPVM<br>*Explain why this information is not reported/not relevant.*
A copy of any certificate(s)	☒ Yes ☐ No	The certification report is given in supplementary materials<br>*Explain why this information is not reported/not relevant.*

8. Statistics

Number of solar cells tested	☒ Yes ☐ No	18 cells are used for statistic analysis<br>*Explain why this information is not reported/not relevant.*
Statistical analysis of the device performance	☒ Yes ☐ No	Statistical analysis of the device performance is given in supplementary figure 2 and supplementary figure 21<br>*Explain why this information is not reported/not relevant.*

9. Long-term stability analysis

Type of analysis, bias conditions and environmental conditions	☐ Yes ☒ No	*Provide a description of the type of analysis, bias conditions and environmental conditions (e.g. illumination type, temperature, atmosphere humidity, encapsulation method, preconditioning temperature, bias) for each long-term stability analysis carried out; see ref. 7 and 8 for details.*<br>No stability problem has been reported in kesterite solar cells
